# Differential Longitudinal Associations Between Domains of Cognitive Function and Physical Function: A 20-Year Follow-Up Study

**DOI:** 10.1093/geronb/gbad156

**Published:** 2023-10-18

**Authors:** Giovanni Sala, Yukiko Nishita, Chikako Tange, Shu Zhang, Fujiko Ando, Hiroshi Shimokata, Rei Otsuka, Hidenori Arai

**Affiliations:** Department of Psychology, Institute of Population Health, University of Liverpool, Liverpool, UK; Department of Epidemiology of Aging, Research Institute, National Center for Geriatrics and Gerontology, Obu, Aichi, Japan; Department of Epidemiology of Aging, Research Institute, National Center for Geriatrics and Gerontology, Obu, Aichi, Japan; Department of Epidemiology of Aging, Research Institute, National Center for Geriatrics and Gerontology, Obu, Aichi, Japan; Department of Epidemiology of Aging, Research Institute, National Center for Geriatrics and Gerontology, Obu, Aichi, Japan; Department of Epidemiology of Aging, Research Institute, National Center for Geriatrics and Gerontology, Obu, Aichi, Japan; Faculty of Health and Medical Sciences, Aichi Shukutoku University, Nagakute, Aichi, Japan; Department of Epidemiology of Aging, Research Institute, National Center for Geriatrics and Gerontology, Obu, Aichi, Japan; Graduate School of Nutritional Sciences, Nagoya University of Arts and Sciences, Nisshin, Aichi, Japan; Department of Epidemiology of Aging, Research Institute, National Center for Geriatrics and Gerontology, Obu, Aichi, Japan; National Center for Geriatrics and Gerontology, Obu, Aichi, Japan; (Psychological Sciences Section)

**Keywords:** Cognition, Dementia, Frailty, Quantitative Methods

## Abstract

**Objectives:**

Cognitive and physical functions are both associated with disability and death. Recent studies have addressed the relationship between cognitive declines and physical declines; however, whether various facets of cognition are diversely associated with specific physical functions is yet to be ascertained. The present work examines the longitudinal associations between fluid and crystallized cognitive functions (*Gf* and *Gc*) and physical functions.

**Methods:**

The sample consisted of 863 community-dwelling older adults (baseline age 60–79 years) from the National Institute for Longevity Sciences-Longitudinal Study of Aging. The participants were tested on a set of *Gf* and *Gc* tests and physical tests (grip strength and gait speed). We ran a series of Multivariate Latent Growth Curve models. Specifically, we tested the relationship between cognitive and physical functions in terms of baseline performance (intercept) and rate of change (slope).

**Results:**

The slope–slope correlations between *Gf* and physical function were large (grip strength *r* = 0.64 and gait speed *r* = 0.68, *p*s < .001). By contrast, the slope correlations between *Gc* and physical functions were weak (*r*s ≤ 0.31) and barely or marginally significant (*p*s ≤ .06).

**Discussion:**

The results show that distinct domains of cognitive functions have different associations with physical functions. Namely, the aging-associated declines in the tested physical functions are robustly correlated with the declines in *Gf*, but are only weakly correlated with the declines in *Gc*. Therefore, *Gc* measures may be poor proxies for the patient’s frailty and should be considered with caution in clinical assessment.

Preserving cognitive function and physical function is paramount to the quality of life in older adults. Cognitive and physical health is essential for the senior citizen’s daily activities and contributes to alleviating the societal burden posed by long-term care. Investigating aging-associated cognitive and physical declines throughout the lifespan is thus a priority for geriatrics research (Hoogendijk et al., 2019; Livingston et al., 2020; Lövdén et al., 2020).

It has been suggested that cognitive and noncognitive aging-associated declines stem from the same seed. For example, the deterioration of the physiology of the entire organism due to senescence may be responsible for the decline of a wide variety of abilities. Such explanations involving a common factor subtending the downturn of multiple intellective and nonintellective skills are often subsumed under the umbrella term *common-cause hypothesis* (Christensen et al., 2001).

The study of the relationship between cognitive declines and physical declines is a particular case of the common-cause hypothesis. Overall, cognitive aging-associated declines often correlate with physical declines (Hackett et al., 2018; Wu et al., 2015; Zammit et al., 2021). The combination of cognitive and physical declines, usually referred to as *cognitive frailty* or physio-cognitive decline syndrome, has been suggested to be a potential correlate of aging-related neurodegenerative processes (Kelaiditi et al., 2013; Liu et al., 2020).

However, cognition is not a unidimensional trait. General cognitive ability consists of two conceptually, albeit correlated, distinct constructs (McGrew et al., 1997). Crystallized functions (hereafter *Gc*) pertain to those cognitive abilities one gains and develops in their social environment. *Gc* thus encompasses skills such as literacy, numeracy, and domain-specific knowledge in general. By contrast, fluid functions (hereafter *Gf*) refer to cognitive abilities such as processing speed and working memory.

*Gc* and *Gf* exhibit different trajectories across the lifespan. *Gc* appears to be less affected by age compared to *Gf* (Rönnlund et al., 2005; Schaie, 1994). Conversely, *Gf* often shows steeper aging-associated declines (Nishita et al., 2013). Additionally, *Gf* seems to be less malleable to training than *Gc* (Gobet & Sala, 2023; Ritchie et al., 2015; Simons et al., 2016). It is thus essential to discriminate between *Gf* and *Gc* when examining the relationship between physical declines and cognitive declines. Nonetheless, empirical studies usually do not distinguish the domains of cognitive function associate with physical declines (Bai et al., 2021; Chen et al., 2018; Chou et al., 2019; Huang et al., 2020; Wu et al., 2015).

Furthermore, investigating multivariate aging-associated declines, such as the relation between cognitive function and physical function trajectories in old age, poses a methodological challenge. To reach a statistical power sufficient to detect deviations in longitudinal changes, three elements are paramount: numerous assessment time points (to reduce measurement error), large samples (e.g., *N* > 500), and extensive time spans (e.g., decades) that permit recording of significant aging-related declines (Brandmaier et al., 2018; Hertzog et al., 2006). All these conditions have been rarely (if ever) met (Béland et al., 2018; Lu et al., 2021; Zaninotto et al., 2018).

The present work fills these gaps. We here examine the differential longitudinal relationships between physical function and domains of cognitive functions (*Gf* vs *Gc*). Importantly, we employ data that meet all the above necessary requirements to obtain an adequate statistical power.

## Method

### Participants

The data were a subset of the National Institute for Longevity Sciences-Longitudinal Study of Aging (NILS-LSA; Shimokata et al., 2000). The NILS-LSA is a population-based prospective cohort study about aging and age-related diseases. The participants (*N* = 2,267 at baseline) were sex- and age-stratified random samples of Japanese community-dwelling adults aged from 40 to 79 years at baseline (Wave 1: 1997–2000). They were followed up every 2–6 years (Wave 2: 2000–2002, Wave 3: 2002–2004, Wave 4: 2004–2006, Wave 5: 2006–2008, Wave 6: 2008–2010, Wave 7: 2010–2012, Wave 8: 2013–2016, Wave 9: 2018–2022). The study was approved by the Ethics Committee of Human Research at the National Center for Geriatrics and Gerontology, Japan (No. 899-6; 1351-2). The participants provided written informed consent.

This study included those participants (*N* = 863) that satisfied the following conditions: (a) at least one data point in addition to baseline assessment (*N* = 360 excluded); (b) no history of dementia at baseline (Wave 1; *N* = 4 excluded) assessment; (c) no missing data in any of the covariates at baseline assessment (*N* = 11 excluded); and (d) older than 60 years old (*N* = 1,029 excluded). The ratio of participants older than 60 years of age with only one data point (*N* = 251) to the total population over 60 (i.e., *N* = 863 plus *N* = 251) was 0.225 (or 22.5%).

The mean time between first and last assessments was 10.17 (standard deviation [*SD*] = 6.47). The mean number of assessments was 3.68 (*SD* = 2.48).

### Model

#### Variables

##### Cognitive assessment

Cognitive performance was assessed with three subscales of the Japanese version of the Wechsler Adult Intelligence Scale Revised Short Form (Wechsler, 1981): the *Information* test, the *Similarities* test, and the *Digit Symbol Substitution Test* (DSST). The *Information* test assesses declarative knowledge of commonly known facts. Participants are asked to answer general knowledge questions covering people, places, and events (29 items, score range 0–29). The *Similarities* test assesses logical, abstract thinking by asking participants to state how two things are similar (14 items, score range 0–28). Finally, the DSST assesses one’s processing speed. Participants need to write as many symbols as possible that correspond to a given number in 90 s (score range 0–93). In the three tests, higher scores indicate better performance. A composite score (average of *z*-scores) of *Information* and *Similarities* served as a proxy for *Gc*; the DSST (*z*-score) was utilized as a proxy for *Gf*.

##### Physical assessment

Physical function was assessed with the *gait speed* test and the *grip strength* test, which are proxies for lower-body and upper-body strength, respectively. Participants were told to walk on an 11-m straight walkway at a comfortable speed (including 1 m for acceleration and deceleration). Light sensors (Yagami, Aichi, Japan) were used to record the time taken to walk 10 m at the start and end points. *Gait speed* was measured in meters per second (m/s). *Grip strength* was measured in kilograms (kg) with a handgrip dynamometer (Takei Co., Niigata, Japan; Kozakai et al., 2016). The participants were asked to stand and extend their elbows to hold the dynamometer. Two trials per hand were ran, and the maximum value was employed in the analysis.

##### Intercepts and slopes

The latent intercepts and slopes were estimated from the observed variables (i.e., *Gc*, *Gf*, *gait speed*, and *grip strength*). The mean slopes indicated whether cognitive function increased or decreased on average over time. The mean intercepts represented the scaled estimated average baseline performance. The intercept variance and the slope variance showed between-subject differences at baseline and in aging-associated change, respectively.

##### Covariates

The models’ estimates were adjusted by including several numeric and binary covariates. These covariates included the participant’s education in years (numeric), baseline age in years (numeric), sex (male or female), living arrangements (living alone or with others), previously or currently smoking (yes or no), apolipoprotein E (APOE) genotype (carrying the ε4 allele or not), and having a history (present or none) of stroke, diabetes, or hypertension.

### Statistical Analysis

**Figure 1.**
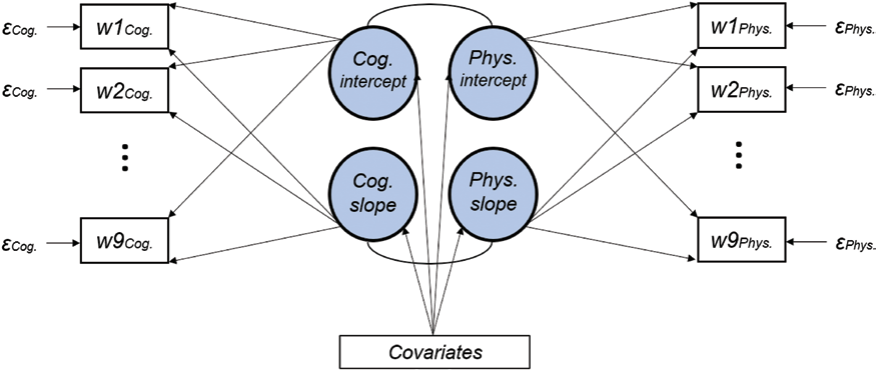
The schematic of the Multivariate Latent Growth Model. The four latent factors (circles), the two intercept and the slope, predicted the observed cognitive (*cog*) variables and physical (*phy*) variables (*t*s; waves from 3 to 8 were omitted for the sake of clarity). Residuals (ɛs) were constrained to be equal across waves.

A set of Multivariate Latent Growth Curve (MLGC) models were run (Robitaille et al., 2012). Two latent intercepts and two latent slopes were estimated for all the combinations of cognitive (*Gf* vs *Gc*) and physical (*gait speed* vs *grip strength*) variables (2 × 2 design for a total of four models). The factor loadings were fixed to 1 for the intercepts. Age-associated declines were allowed to be nonlinear. Thus, the slope loadings were freely estimated (except the factor loadings of the first wave and last wave, which were fixed to 0 and 1 to set the scale). A set of time-invariant covariates were added to the model as predictors of the latent variables. The residuals were constrained to be equal across the waves to facilitate model convergence. The slopes and the intercepts were allowed to correlate. The numeric variables were all centered and scaled. Figure 1 depicts the whole model.

As expected, attrition (i.e., the loss of participants) over the nine waves of the survey was substantial (from Wave 2 to Wave 9, 822 [95%], 679 [79%], 588 [68%], 488 [57%], 410 [48%], 339 [39%], 262 [30%], and 140 [16%] participants, respectively). Full Information Maximum Likelihood (FIML) was employed to handle missing data due to attrition. Unlike listwise deletion, FIML uses all the available data, including cases with missing values, to estimate model parameters. FIML thus maximizes statistical power, leading to more efficient and precise parameter estimates. FIML is employed when data are missing completely at random (MCAR; i.e., when missingness does not depend on any systematic factors) and when data are missing at random (MAR; i.e., when missingness depends on any systematic factors that are included in the model, as baseline age and baseline cognitive and physical frailty). We thus assumed data to be mostly MCAR and MAR. Nonetheless, it is worth mentioning that, as in any longitudinal survey, there may be unobserved variables affecting the participants’ dropout patterns.

We ran the analyses with the *lavaan R* package (R Core Team, 2021; Rosseel, 2012). All the details regarding the models’ parameters, fit indexes, correlation matrices, and residual matrices are reported at https://osf.io/3b4uh/.

## Results

The baseline descriptive statistics are summarized in Table 1. Means and *SD*s are reported for the numeric variables. Counts and percentages are reported for the binary covariates.

**Table 1.** The Baseline Descriptive Statistics of the Participants

Variable	Mean	*SD*	*N*	%
Age (year)	68.64	5.38		
Education (year)	10.68	2.64		
Information (point)	13.35	5.59		
Similarities (point)	11.48	5.53		
DSST (point)	42.48	11.22		
Grip strength (kg)	29.47	9.06		
Gait speed (m/s)	1.26	0.19		
Males			409	47.4
Living alone			64	7.4
Smoking			379	43.9
APOE4 carrier			171	19.8
Stroke			33	3.8
Hypertension			316	36.6
Diabetes			102	11.8

*Notes*: DSST = Digit Symbol Substitution Test; *SD* = standard deviation.

The goodness of fit indexes (the Comparative Fit Index [CFI], the Standardized Root Mean Square Residual [SRMR], and the Root Mean Square Error of Approximation [RMSEA]) indicated an acceptable fit in all the models (all CFIs ≥ 0.96, SRMRs ≤ 0.08, and RMSEAs ≤ 0.04). The models’ estimates can be deemed trustworthy (for the details, see Supplementary Table S1). Furthermore, the models, whose slopes’ factor loadings were allowed to estimate nonlinear longitudinal change, exhibited a better fit compared to linear models (*p*s < 10^−11^; Satorra–Bentler test for nested models). Table 2 reports the factor loadings sorted by type of outcome.

**Table 2.** The Slope Factor Loadings Sorted by Latent Factor and Model

Model	Cognitive slope loadings	Physical slope loadings
*Gf* & *gait speed*	[0, 0.07, 0.07, 0.12, 0.21, 0.27, 0.37, 0.59, 1]	[0, 0.01, 0.06, 0.14, 0.18, 0.29, 0.48, 0.69, 1]
*Gf* & *grip strength*	[0, 0.07, 0.06, 0.11, 0.20, 0.26, 0.36, 0.59, 1]	[0, 0.20, 0.23, 0.41, 0.48, 0.62, 0.71, 0.67, 1]
*Gc* & *gait speed*	[0, −0.01, 0.14, 0.20, 0.37, 0.38, 0.48, 0.69, 1]	[0, 0.00, 0.05, 0.16, 0.20, 0.30, 0.50, 0.72, 1]
*Gc* & *grip strength*	[0, −0.02, 0.13, 0.19, 0.34, 0.34, 0.45, 0.68, 1]	[0, 0.21, 0.24, 0.43, 0.49, 0.64, 0.73, 0.68, 1]

The decrease in *Gf* over the two decades of the survey was large. The standardized mean slopes (i.e., the mean change over the whole survey in *SD*s) were −1.33 and −1.41, when estimated with gait speed and grip strength, respectively. The decrease in *Gc* was less pronounced, but still substantial (the standardized mean slopes were −0.88 and −0.93). Analogously, both gait speed and grip strength exhibited steep declines in all the four models (−1.33, −2.11 and −1.35, −2.14, in the *Gf* and *Gc* models, respectively). The comparability between standardized mean sloped was possible thanks to the consistent standardization of the slope factor loadings (i.e., Wave 1 and Wave 9 factor loadings are fixed to 0 and 1 in all the models).

The model-estimated correlations between the latent slopes of the two measures of physical functions and *Gf* (0.68 and 0.64 for gait speed and grip strength, respectively; both *p*s < .001) were greater than the ones with *Gc* (0.31 and 0.28; *p* = .046 and *p* = .057). That is, *Gf* declines shared a much larger amount of variance with physical declines than *Gc* declines (see also Supplementary Figure S1). The intercept–intercept correlations were small or close to zero (*r*s ≤ 0.16; Table 3). Supplement 2 reports all the models’ parameters in detail.

**Table 3.** The Latent Factors’ Correlations Sorted by Model

Model	Coefficient	Estimate	SEM	*p* Value	Std. Est
*Gf* & *gait speed*	intercept–intercept	0.08	0.02	<.001	0.16
	slope–slope	0.49	0.13	<.001	0.68
*Gf & grip strength*	intercept–intercept	0.05	0.02	.001	0.12
	slope–slope	0.26	0.07	<.001	0.64
*Gc* & *gait speed*	intercept–intercept	0.07	0.02	<.001	0.16
	slope–slope	0.10	0.05	.046	0.31
*Gc* & *grip strength*	intercept–intercept	0.02	0.01	.147	0.05
	slope–slope	0.06	0.03	.057	0.28

*Notes*: Estimate = unstandardized model coefficient (covariance); *p* Value = statistical significance; SEM = standard error of mean; Std. Est = standardized model coefficient (correlation).

Finally, alternative ways to address longitudinal attrition did not produce meaningfully different results. Partial listwise deletion (e.g., keeping only those participants who took part in at least five waves; *N* = 491), instead of FIML, provided the same pattern of results. The correlations between *Gf* declines and physical declines were substantial (*r*s = 0.65; *p*s < .001). Conversely, the correlations between *Gc* declines and physical declines were weak (*r*s = 0.27; *p*s > .05). These results upheld the idea that there was a decoupling between *Gf* and *Gc* declines irrespective of the mechanisms driving the participants’ dropouts.

## Discussion

This work has examined the relationship between aging-associated cognitive declines and physical declines. We have collected data from a sample of Japanese older adults over nine waves spanning approximately 20 years. Two dimensions of cognitive function and two dimensions of physical function have been evaluated in four MLGC models and included a set of covariates to control for potential confounding effects. Several studies have been conducted to explore the relationship between physical and cognitive function in the older adults. For instance, Huang et al. (2020) have shown that mobility subtype frailty (characterized by low grip strength and low walking speed) is associated with decreased DSST scores. However, like other similar investigation, this study does not estimate the correlation between trajectories of physical and cognitive functions, implements a relatively short follow-up (which reduces the statistical power necessary to detect longitudinal change), and focuses solely on one domain of cognitive function (i.e., without collecting any *Gc* measures).

The results in present study show a pronounced difference between *Gf*- and *Gc*-related outcomes. While the correlation between aging-associated physical declines and *Gf* declines (slope–slope correlations) is substantial, *Gc* declines are only loosely related to physical declines. Interestingly, this pattern of results is independent of the type of physical function tested (lower- or upper-body strength). Thus, these outcomes are unlikely to be the mere byproduct of one’s physical inability to perform complex paper-and-pencil cognitive tests (e.g., the DSST). Furthermore, baseline cognitive and physical performances are, at most, only barely related (near-zero intercept–intercept correlations). It is therefore reasonable to suppose that physical prowess (or the lack thereof) can hardly be a serious confounding factor.

In brief, aging-related physical and cognitive declines can be reasonably seen as two dimensions of a more general phenomenon, as suggested in the cognitive frailty framework (Cesari et al., 2020). Nevertheless, the relationship exhibits some specifics of interest. Namely, it seems to be more appreciable in decline rates rather than baseline performances and, most notably, when fluid cognitive functions (*Gf*) rather than crystallized cognitive functions (*Gc*) are involved.

The decoupling of *Gf* and *Gc* declines in their relationship with physical declines is probably the consequence of the nature of these constructs. Along with physical declines, *Gf* declines express a more general phenomenon, that is, one’s overall aging-associated decline (as theorized in the cognitive frailty framework and similar frameworks). In fact, *Gf* is a measure of core cognitive mechanisms. The deterioration of such mechanisms is likely to share the same causes with physical declines (e.g., neurodegenerative processes). By contrast, *Gc*, which consists of domain-specific knowledge acquired throughout the lifespan, does not produce the same pattern of results.

These findings bear practical implications concerning clinical assessment with geriatric patients. *Gf* measures should be preferred over *Gc* measures when evaluating the patient’s frailty. In fact, if cognitive frailty is defined as the combination of cognitive and physical declines, and *Gc* decline does not go along with physical decline, *Gc* is, ipso facto, not a reliable proxy for cognitive frailty. This does not necessarily imply that *Gc*-related performance examination is of no use in older patients. Nonetheless, *Gc* appears to be a weak proxy for aging-associated general declines. Thus, the patient’s good performance in *Gc* tests should not be regarded as a sufficient criterion to exclude the possibility of frailty-associated conditions.

Finally, it is worth mentioning the present study’s main limitations. First, *Gf* and *Gc* were assessed with only one and two tests, respectively. We thus suggest testing the relationship between physical and cognitive declines by employing a broader range of cognitive tests (e.g., nonverbal and verbal working memory). Moreover, this sample consisted of relatively healthier older adults compared to the general population. For example, the participants autonomously traveled to the venue of the data collection, which required a certain degree of functional independence. Although the stratified random sampling probably mitigates this potential issue, examining the relationship between physical and cognitive declines in less active older adults would be a valid extension of our findings.

## Conclusion

Declines in fluid cognitive functions (*Gf*) are strongly correlated with both gait speed and grip strength declines (slope–slope correlations). Conversely, declines in crystallized cognitive functions (*Gc*) are weakly correlated with declines in physical performance. While corroborating the cognitive frailty theory, these results highlight the need to distinguish between fluid and crystallized cognitive declines in connection with physical declines.

## Supplementary Material

gbad156_suppl_Supplementary_Material

gbad156_suppl_Supplementary_Data

## Data Availability

The data and the R codes to reproduce the results are publicly available at https://osf.io/3b4uh/. This study was not preregistered.
