# Supplementary material for: Differential Longitudinal Associations Between Domains of Cognitive Function and Physical Function: A 20-Year Follow-Up Study
Source: J Gerontol B Psychol Sci Soc Sci. 2023 Oct 18;79(1):gbad156. doi: 10.1093/geronb/gbad156 (PMC10745265; doi:10.1093/geronb/gbad156)
Supplement: gbad156_suppl_Supplementary_Material [file gbad156_suppl_supplementary_material.docx]

# Supplement 1

## Supplemental Figures


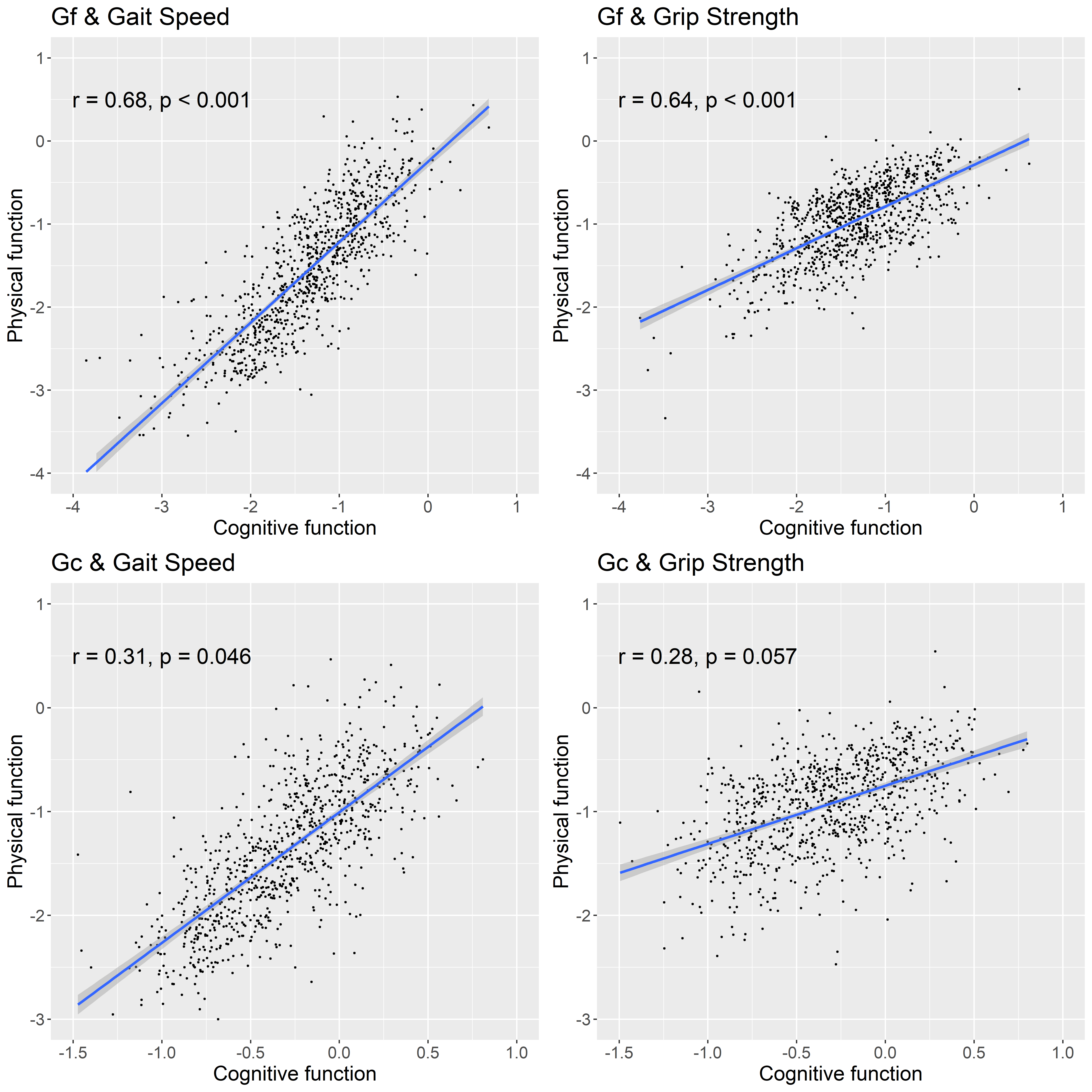


**Figure S1**. Scatterplots of the participants’ model-estimated slopes for cognitive function (x-axis) and physical function (y-axis). The fitted lines indicate the correlation between the slopes. The gray area represents 95% CIs.

## Fit Indexes

Table S1

*The models’ goodness of fit indexes*

| Model | chisq | df | cfi | srmr | rmsea |
| --- | --- | --- | --- | --- | --- |
| Gf & Gait Speed | 721.98 | 285 | 0.96 | 0.08 | 0.04 |
| Gf & Grip Strength | 769.64 | 285 | 0.97 | 0.05 | 0.04 |
| Gc & Gait Speed | 661.87 | 285 | 0.96 | 0.04 | 0.04 |
| Gc & Grip Strength | 642.42 | 285 | 0.98 | 0.03 | 0.04 |

## Power Analysis

alpha <- .05

semPower(type = "post-hoc", effect = .05, effect.measure = "RMSEA",
alpha = alpha, N = nrow(data_final), df = inspect(list_models[[1]], "fit.measures")["df"])

## $type
## [1] "post-hoc"
##
## $alpha
## [1] 0.05
##
## $beta
## [1] 1.881182e-41
##
## $power
## [1] 1
##
## $impliedAbratio
## [1] 2.657904e+39
##
## $ncp
## [1] 614.175
##
## $fmin
## [1] 0.7125
##
## $effect
## [1] 0.05
##
## $effect.measure
## [1] "RMSEA"
##
## $N
## [1] 863
##
## $df
## df
## 285
##
## $p
## NULL
##
## $chiCrit
## [1] 325.3741
##
## $rmsea
## df
## 0.05
##
## $mc
## [1] 0.7002975
##
## $gfi
## NULL
##
## $agfi
## NULL
##
## $srmr
## NULL
##
## $cfi
## NULL
##
## attr(,"class")
## [1] "semPower.postHoc"

## Correlation Matrices

## Gf1 Gf2 Gf3 Gf4 Gf5 Gf6 Gf7 Gf8 Gf9
## Gf1 1.000
## Gf2 0.880 1.000
## Gf3 0.880 0.884 1.000
## Gf4 0.878 0.885 0.885 1.000
## Gf5 0.869 0.881 0.881 0.887 1.000
## Gf6 0.862 0.877 0.877 0.885 0.895 1.000
## Gf7 0.845 0.865 0.864 0.877 0.892 0.899 1.000
## Gf8 0.800 0.828 0.828 0.846 0.873 0.885 0.904 1.000
## Gf9 0.707 0.746 0.746 0.773 0.814 0.834 0.868 0.915 1.000
## sc_velo1 0.320 0.336 0.336 0.348 0.365 0.373 0.387 0.406 0.417
## sc_velo2 0.322 0.339 0.339 0.350 0.368 0.376 0.390 0.409 0.422
## sc_velo3 0.333 0.352 0.352 0.365 0.384 0.394 0.410 0.433 0.449
## sc_velo4 0.351 0.373 0.373 0.388 0.411 0.423 0.443 0.471 0.494
## sc_velo5 0.358 0.381 0.381 0.397 0.422 0.434 0.455 0.487 0.512
## sc_velo6 0.372 0.399 0.398 0.417 0.446 0.460 0.484 0.521 0.554
## sc_velo7 0.390 0.420 0.420 0.442 0.476 0.493 0.523 0.569 0.612
## sc_velo8 0.398 0.433 0.432 0.457 0.495 0.514 0.548 0.601 0.653
## sc_velo9 0.401 0.438 0.438 0.465 0.507 0.528 0.566 0.625 0.686
## Education 0.466 0.458 0.458 0.450 0.434 0.425 0.405 0.362 0.289
## Age -0.453 -0.472 -0.472 -0.484 -0.503 -0.511 -0.525 -0.541 -0.544
## Gender -0.033 -0.027 -0.027 -0.023 -0.016 -0.012 -0.004 0.009 0.028
## Living_alone -0.013 -0.020 -0.020 -0.025 -0.033 -0.037 -0.045 -0.059 -0.076
## Smoking 0.012 0.008 0.008 0.005 0.000 -0.003 -0.009 -0.018 -0.031
## Apoe4 -0.018 -0.026 -0.025 -0.031 -0.040 -0.044 -0.053 -0.068 -0.086
## stroke1 -0.112 -0.115 -0.115 -0.116 -0.119 -0.120 -0.121 -0.121 -0.117
## h_t1 -0.073 -0.072 -0.072 -0.070 -0.068 -0.066 -0.063 -0.056 -0.044
## dm1 -0.059 -0.062 -0.062 -0.065 -0.069 -0.071 -0.075 -0.080 -0.084
## i_Gf 0.938 0.938 0.938 0.935 0.926 0.919 0.901 0.852 0.753
## i_sc_velo 0.378 0.398 0.398 0.411 0.431 0.441 0.457 0.480 0.493
## s_Gf 0.201 0.263 0.263 0.309 0.383 0.422 0.493 0.616 0.764
## s_sc_velo 0.316 0.358 0.358 0.388 0.437 0.462 0.507 0.581 0.663
## sc_vl1 sc_vl2 sc_vl3 sc_vl4 sc_vl5 sc_vl6 sc_vl7 sc_vl8 sc_vl9
## Gf1
## Gf2
## Gf3
## Gf4
## Gf5
## Gf6
## Gf7
## Gf8
## Gf9
## sc_velo1 1.000
## sc_velo2 0.716 1.000
## sc_velo3 0.719 0.720 1.000
## sc_velo4 0.719 0.721 0.730 1.000
## sc_velo5 0.718 0.720 0.731 0.746 1.000
## sc_velo6 0.710 0.713 0.728 0.750 0.758 1.000
## sc_velo7 0.687 0.690 0.712 0.745 0.757 0.784 1.000
## sc_velo8 0.654 0.658 0.685 0.727 0.744 0.780 0.825 1.000
## sc_velo9 0.605 0.610 0.642 0.694 0.715 0.761 0.823 0.864 1.000
## Education 0.225 0.225 0.226 0.225 0.224 0.220 0.211 0.200 0.183
## Age -0.398 -0.401 -0.424 -0.462 -0.477 -0.512 -0.559 -0.591 -0.615
## Gender -0.147 -0.148 -0.149 -0.150 -0.150 -0.149 -0.146 -0.140 -0.131
## Living_alone -0.066 -0.067 -0.071 -0.077 -0.079 -0.085 -0.092 -0.098 -0.101
## Smoking 0.035 0.035 0.038 0.042 0.044 0.048 0.054 0.058 0.062
## Apoe4 -0.014 -0.014 -0.015 -0.018 -0.019 -0.021 -0.025 -0.027 -0.030
## stroke1 -0.139 -0.139 -0.142 -0.146 -0.147 -0.149 -0.150 -0.148 -0.143
## h_t1 -0.101 -0.101 -0.105 -0.110 -0.112 -0.117 -0.122 -0.124 -0.125
## dm1 -0.070 -0.071 -0.078 -0.089 -0.093 -0.104 -0.119 -0.131 -0.141
## i_Gf 0.341 0.343 0.355 0.374 0.381 0.397 0.415 0.425 0.427
## i_sc_velo 0.846 0.847 0.850 0.850 0.848 0.840 0.812 0.773 0.715
## s_Gf 0.327 0.332 0.363 0.416 0.438 0.489 0.562 0.618 0.668
## s_sc_velo 0.258 0.265 0.312 0.392 0.425 0.504 0.621 0.716 0.807
## Eductn Age Gender Lvng_l Smokng Apoe4 strok1 h_t1 dm1
## Gf1
## Gf2
## Gf3
## Gf4
## Gf5
## Gf6
## Gf7
## Gf8
## Gf9
## sc_velo1
## sc_velo2
## sc_velo3
## sc_velo4
## sc_velo5
## sc_velo6
## sc_velo7
## sc_velo8
## sc_velo9
## Education 1.000
## Age -0.230 1.000
## Gender -0.191 -0.019 1.000
## Living_alone 0.007 0.146 0.183 1.000
## Smoking 0.120 -0.004 -0.646 -0.086 1.000
## Apoe4 -0.007 0.035 -0.006 0.015 -0.023 1.000
## stroke1 -0.017 0.129 -0.141 -0.056 0.095 -0.023 1.000
## h_t1 -0.002 0.108 0.049 0.051 -0.013 -0.070 0.087 1.000
## dm1 -0.032 0.036 -0.103 -0.021 0.074 0.025 0.021 0.102 1.000
## i_Gf 0.497 -0.483 -0.035 -0.014 0.013 -0.019 -0.119 -0.078 -0.062
## i_sc_velo 0.266 -0.470 -0.174 -0.079 0.041 -0.016 -0.164 -0.119 -0.083
## s_Gf -0.029 -0.389 0.078 -0.106 -0.062 -0.117 -0.069 0.007 -0.072
## s_sc_velo 0.069 -0.573 -0.063 -0.094 0.063 -0.033 -0.090 -0.097 -0.151
## i_Gf i_sc_v s_Gf s_sc_v
## Gf1
## Gf2
## Gf3
## Gf4
## Gf5
## Gf6
## Gf7
## Gf8
## Gf9
## sc_velo1
## sc_velo2
## sc_velo3
## sc_velo4
## sc_velo5
## sc_velo6
## sc_velo7
## sc_velo8
## sc_velo9
## Education
## Age
## Gender
## Living_alone
## Smoking
## Apoe4
## stroke1
## h_t1
## dm1
## i_Gf 1.000
## i_sc_velo 0.403 1.000
## s_Gf 0.214 0.386 1.000
## s_sc_velo 0.336 0.305 0.721 1.000
## Gf1 Gf2 Gf3 Gf4 Gf5 Gf6 Gf7 Gf8 Gf9
## Gf1 1.000
## Gf2 0.879 1.000
## Gf3 0.879 0.883 1.000
## Gf4 0.878 0.883 0.883 1.000
## Gf5 0.871 0.881 0.880 0.885 1.000
## Gf6 0.865 0.877 0.877 0.883 0.893 1.000
## Gf7 0.850 0.866 0.866 0.875 0.890 0.896 1.000
## Gf8 0.807 0.831 0.830 0.846 0.872 0.883 0.901 1.000
## Gf9 0.720 0.754 0.753 0.775 0.815 0.834 0.866 0.914 1.000
## sc_grip1 0.180 0.176 0.176 0.173 0.166 0.162 0.153 0.134 0.104
## sc_grip2 0.203 0.203 0.203 0.203 0.201 0.200 0.197 0.187 0.167
## sc_grip3 0.207 0.207 0.207 0.207 0.206 0.205 0.203 0.194 0.175
## sc_grip4 0.227 0.232 0.232 0.235 0.239 0.241 0.243 0.244 0.236
## sc_grip5 0.234 0.241 0.240 0.244 0.251 0.254 0.258 0.261 0.257
## sc_grip6 0.249 0.259 0.259 0.265 0.276 0.281 0.289 0.301 0.306
## sc_grip7 0.258 0.270 0.270 0.278 0.292 0.299 0.310 0.327 0.338
## sc_grip8 0.254 0.265 0.265 0.272 0.285 0.291 0.301 0.315 0.323
## sc_grip9 0.280 0.299 0.298 0.311 0.334 0.345 0.366 0.398 0.428
## Education 0.465 0.457 0.458 0.451 0.437 0.428 0.410 0.368 0.298
## Age -0.454 -0.471 -0.470 -0.481 -0.499 -0.508 -0.521 -0.539 -0.544
## Gender -0.036 -0.027 -0.027 -0.021 -0.007 -0.001 0.013 0.037 0.070
## Living_alone -0.015 -0.020 -0.020 -0.023 -0.029 -0.033 -0.039 -0.050 -0.063
## Smoking 0.016 0.008 0.008 0.003 -0.008 -0.013 -0.024 -0.043 -0.068
## Apoe4 -0.017 -0.026 -0.026 -0.032 -0.043 -0.049 -0.060 -0.080 -0.104
## stroke1 -0.113 -0.115 -0.115 -0.116 -0.117 -0.118 -0.118 -0.117 -0.111
## h_t1 -0.074 -0.071 -0.071 -0.069 -0.064 -0.062 -0.057 -0.046 -0.031
## dm1 -0.059 -0.061 -0.061 -0.062 -0.065 -0.066 -0.068 -0.071 -0.072
## i_Gf 0.938 0.938 0.938 0.936 0.928 0.922 0.906 0.860 0.767
## i_sc_grip 0.185 0.180 0.180 0.177 0.170 0.166 0.157 0.138 0.106
## s_Gf 0.211 0.268 0.265 0.303 0.376 0.413 0.483 0.607 0.755
## s_sc_grip 0.103 0.139 0.137 0.162 0.209 0.233 0.279 0.360 0.458
## sc_gr1 sc_gr2 sc_gr3 sc_gr4 sc_gr5 sc_gr6 sc_gr7 sc_gr8 sc_gr9
## Gf1
## Gf2
## Gf3
## Gf4
## Gf5
## Gf6
## Gf7
## Gf8
## Gf9
## sc_grip1 1.000
## sc_grip2 0.944 1.000
## sc_grip3 0.942 0.946 1.000
## sc_grip4 0.922 0.937 0.938 1.000
## sc_grip5 0.912 0.931 0.933 0.940 1.000
## sc_grip6 0.884 0.912 0.915 0.931 0.935 1.000
## sc_grip7 0.860 0.894 0.898 0.921 0.927 0.935 1.000
## sc_grip8 0.871 0.903 0.906 0.926 0.931 0.936 0.936 1.000
## sc_grip9 0.767 0.819 0.826 0.867 0.880 0.905 0.917 0.912 1.000
## Education 0.208 0.208 0.208 0.206 0.205 0.200 0.197 0.198 0.180
## Age -0.203 -0.242 -0.247 -0.283 -0.295 -0.322 -0.339 -0.331 -0.383
## Gender -0.788 -0.771 -0.768 -0.741 -0.729 -0.697 -0.672 -0.684 -0.579
## Living_alone -0.157 -0.159 -0.159 -0.159 -0.159 -0.157 -0.155 -0.156 -0.146
## Smoking 0.504 0.495 0.493 0.477 0.470 0.451 0.436 0.443 0.378
## Apoe4 -0.007 -0.009 -0.009 -0.010 -0.010 -0.011 -0.012 -0.012 -0.013
## stroke1 0.070 0.062 0.061 0.053 0.050 0.042 0.036 0.039 0.018
## h_t1 -0.067 -0.074 -0.075 -0.081 -0.083 -0.087 -0.090 -0.089 -0.095
## dm1 0.009 0.000 -0.001 -0.010 -0.013 -0.020 -0.025 -0.023 -0.039
## i_Gf 0.192 0.217 0.220 0.242 0.250 0.266 0.275 0.271 0.298
## i_sc_grip 0.976 0.967 0.965 0.945 0.935 0.906 0.881 0.893 0.786
## s_Gf -0.028 0.049 0.060 0.136 0.163 0.226 0.268 0.249 0.391
## s_sc_grip -0.525 -0.433 -0.419 -0.322 -0.285 -0.199 -0.138 -0.166 0.051
## Eductn Age Gender Lvng_l Smokng Apoe4 strok1 h_t1 dm1
## Gf1
## Gf2
## Gf3
## Gf4
## Gf5
## Gf6
## Gf7
## Gf8
## Gf9
## sc_grip1
## sc_grip2
## sc_grip3
## sc_grip4
## sc_grip5
## sc_grip6
## sc_grip7
## sc_grip8
## sc_grip9
## Education 1.000
## Age -0.230 1.000
## Gender -0.191 -0.019 1.000
## Living_alone 0.007 0.146 0.183 1.000
## Smoking 0.120 -0.004 -0.646 -0.086 1.000
## Apoe4 -0.007 0.035 -0.006 0.015 -0.023 1.000
## stroke1 -0.017 0.129 -0.141 -0.056 0.095 -0.023 1.000
## h_t1 -0.002 0.108 0.049 0.051 -0.013 -0.070 0.087 1.000
## dm1 -0.032 0.036 -0.103 -0.021 0.074 0.025 0.021 0.102 1.000
## i_Gf 0.496 -0.485 -0.039 -0.016 0.017 -0.018 -0.121 -0.078 -0.062
## i_sc_grip 0.213 -0.208 -0.808 -0.161 0.517 -0.007 0.072 -0.069 0.009
## s_Gf -0.024 -0.389 0.154 -0.087 -0.128 -0.151 -0.057 0.031 -0.053
## s_sc_grip -0.096 -0.218 0.519 0.057 -0.320 -0.007 -0.095 -0.023 -0.074
## i_Gf i_sc_g s_Gf s_sc_g
## Gf1
## Gf2
## Gf3
## Gf4
## Gf5
## Gf6
## Gf7
## Gf8
## Gf9
## sc_grip1
## sc_grip2
## sc_grip3
## sc_grip4
## sc_grip5
## sc_grip6
## sc_grip7
## sc_grip8
## sc_grip9
## Education
## Age
## Gender
## Living_alone
## Smoking
## Apoe4
## stroke1
## h_t1
## dm1
## i_Gf 1.000
## i_sc_grip 0.197 1.000
## s_Gf 0.225 -0.028 1.000
## s_sc_grip 0.109 -0.538 0.635 1.000
## Gc1 Gc2 Gc3 Gc4 Gc5 Gc6 Gc7 Gc8 Gc9
## Gc1 1.000
## Gc2 0.875 1.000
## Gc3 0.873 0.873 1.000
## Gc4 0.870 0.869 0.878 1.000
## Gc5 0.857 0.856 0.873 0.878 1.000
## Gc6 0.856 0.855 0.872 0.878 0.886 1.000
## Gc7 0.846 0.843 0.866 0.873 0.886 0.887 1.000
## Gc8 0.816 0.813 0.845 0.856 0.880 0.881 0.890 1.000
## Gc9 0.765 0.761 0.803 0.820 0.856 0.858 0.874 0.899 1.000
## sc_velo1 0.232 0.230 0.248 0.256 0.272 0.273 0.281 0.294 0.306
## sc_velo2 0.232 0.231 0.249 0.256 0.272 0.273 0.281 0.295 0.307
## sc_velo3 0.235 0.233 0.253 0.261 0.280 0.281 0.290 0.305 0.320
## sc_velo4 0.239 0.237 0.260 0.270 0.292 0.293 0.304 0.324 0.344
## sc_velo5 0.240 0.238 0.263 0.273 0.297 0.298 0.309 0.331 0.352
## sc_velo6 0.241 0.239 0.267 0.279 0.306 0.307 0.320 0.345 0.371
## sc_velo7 0.240 0.237 0.269 0.283 0.315 0.317 0.333 0.364 0.396
## sc_velo8 0.234 0.231 0.267 0.283 0.319 0.321 0.339 0.374 0.412
## sc_velo9 0.224 0.221 0.261 0.277 0.317 0.319 0.340 0.379 0.422
## Education 0.548 0.548 0.552 0.552 0.549 0.549 0.545 0.533 0.508
## Age -0.124 -0.120 -0.170 -0.192 -0.244 -0.247 -0.274 -0.328 -0.391
## Gender -0.199 -0.200 -0.185 -0.177 -0.158 -0.157 -0.145 -0.121 -0.088
## Living_alone 0.052 0.052 0.046 0.042 0.034 0.034 0.029 0.020 0.007
## Smoking 0.064 0.064 0.061 0.059 0.055 0.054 0.052 0.046 0.038
## Apoe4 -0.001 0.000 -0.015 -0.022 -0.038 -0.039 -0.048 -0.066 -0.087
## stroke1 -0.045 -0.044 -0.051 -0.054 -0.061 -0.062 -0.065 -0.072 -0.079
## h_t1 -0.042 -0.041 -0.044 -0.046 -0.048 -0.048 -0.050 -0.052 -0.054
## dm1 -0.032 -0.032 -0.035 -0.037 -0.040 -0.040 -0.042 -0.044 -0.047
## i_Gc 0.936 0.936 0.934 0.930 0.916 0.915 0.904 0.872 0.817
## i_sc_velo 0.274 0.272 0.294 0.302 0.322 0.323 0.332 0.348 0.362
## s_Gc 0.089 0.081 0.170 0.208 0.302 0.306 0.356 0.455 0.573
## s_sc_velo 0.111 0.107 0.152 0.171 0.218 0.220 0.245 0.292 0.348
## sc_vl1 sc_vl2 sc_vl3 sc_vl4 sc_vl5 sc_vl6 sc_vl7 sc_vl8 sc_vl9
## Gc1
## Gc2
## Gc3
## Gc4
## Gc5
## Gc6
## Gc7
## Gc8
## Gc9
## sc_velo1 1.000
## sc_velo2 0.715 1.000
## sc_velo3 0.717 0.717 1.000
## sc_velo4 0.716 0.717 0.725 1.000
## sc_velo5 0.715 0.715 0.725 0.739 1.000
## sc_velo6 0.706 0.707 0.720 0.742 0.749 1.000
## sc_velo7 0.682 0.683 0.702 0.735 0.746 0.771 1.000
## sc_velo8 0.648 0.649 0.674 0.716 0.732 0.766 0.809 1.000
## sc_velo9 0.602 0.603 0.633 0.685 0.704 0.748 0.806 0.846 1.000
## Education 0.224 0.224 0.226 0.228 0.229 0.229 0.225 0.218 0.206
## Age -0.398 -0.400 -0.423 -0.464 -0.479 -0.514 -0.562 -0.597 -0.623
## Gender -0.147 -0.147 -0.149 -0.153 -0.154 -0.155 -0.156 -0.153 -0.148
## Living_alone -0.068 -0.068 -0.071 -0.077 -0.078 -0.083 -0.089 -0.093 -0.095
## Smoking 0.035 0.035 0.039 0.045 0.047 0.053 0.060 0.067 0.072
## Apoe4 -0.014 -0.014 -0.016 -0.019 -0.020 -0.023 -0.027 -0.031 -0.034
## stroke1 -0.138 -0.138 -0.140 -0.143 -0.144 -0.145 -0.145 -0.142 -0.137
## h_t1 -0.099 -0.099 -0.103 -0.111 -0.114 -0.120 -0.129 -0.135 -0.138
## dm1 -0.069 -0.070 -0.076 -0.088 -0.092 -0.103 -0.118 -0.130 -0.141
## i_Gc 0.248 0.248 0.251 0.255 0.256 0.258 0.256 0.250 0.240
## i_sc_velo 0.846 0.846 0.848 0.847 0.845 0.835 0.806 0.766 0.711
## s_Gc 0.231 0.232 0.254 0.294 0.309 0.345 0.397 0.439 0.474
## s_sc_velo 0.200 0.202 0.247 0.332 0.365 0.443 0.563 0.664 0.757
## Eductn Age Gender Lvng_l Smokng Apoe4 strok1 h_t1 dm1
## Gc1
## Gc2
## Gc3
## Gc4
## Gc5
## Gc6
## Gc7
## Gc8
## Gc9
## sc_velo1
## sc_velo2
## sc_velo3
## sc_velo4
## sc_velo5
## sc_velo6
## sc_velo7
## sc_velo8
## sc_velo9
## Education 1.000
## Age -0.230 1.000
## Gender -0.191 -0.019 1.000
## Living_alone 0.007 0.146 0.183 1.000
## Smoking 0.120 -0.004 -0.646 -0.086 1.000
## Apoe4 -0.007 0.035 -0.006 0.015 -0.023 1.000
## stroke1 -0.017 0.129 -0.141 -0.056 0.095 -0.023 1.000
## h_t1 -0.002 0.108 0.049 0.051 -0.013 -0.070 0.087 1.000
## dm1 -0.032 0.036 -0.103 -0.021 0.074 0.025 0.021 0.102 1.000
## i_Gc 0.586 -0.133 -0.213 0.055 0.068 -0.001 -0.048 -0.044 -0.034
## i_sc_velo 0.265 -0.471 -0.173 -0.080 0.042 -0.016 -0.164 -0.117 -0.082
## s_Gc 0.114 -0.578 0.152 -0.070 -0.030 -0.172 -0.085 -0.039 -0.041
## s_sc_velo 0.091 -0.573 -0.081 -0.080 0.078 -0.039 -0.072 -0.116 -0.152
## i_Gc i_sc_v s_Gc s_sc_v
## Gc1
## Gc2
## Gc3
## Gc4
## Gc5
## Gc6
## Gc7
## Gc8
## Gc9
## sc_velo1
## sc_velo2
## sc_velo3
## sc_velo4
## sc_velo5
## sc_velo6
## sc_velo7
## sc_velo8
## sc_velo9
## Education
## Age
## Gender
## Living_alone
## Smoking
## Apoe4
## stroke1
## h_t1
## dm1
## i_Gc 1.000
## i_sc_velo 0.293 1.000
## s_Gc 0.095 0.273 1.000
## s_sc_velo 0.119 0.236 0.514 1.000
## Gc1 Gc2 Gc3 Gc4 Gc5 Gc6 Gc7 Gc8 Gc9
## Gc1 1.000
## Gc2 0.875 1.000
## Gc3 0.874 0.872 1.000
## Gc4 0.871 0.869 0.878 1.000
## Gc5 0.860 0.858 0.874 0.879 1.000
## Gc6 0.860 0.857 0.874 0.878 0.885 1.000
## Gc7 0.848 0.845 0.867 0.874 0.885 0.886 1.000
## Gc8 0.818 0.813 0.845 0.856 0.877 0.878 0.889 1.000
## Gc9 0.763 0.757 0.801 0.816 0.850 0.851 0.870 0.898 1.000
## sc_grip1 0.212 0.213 0.207 0.204 0.197 0.196 0.190 0.176 0.155
## sc_grip2 0.220 0.220 0.218 0.217 0.213 0.213 0.209 0.199 0.184
## sc_grip3 0.221 0.221 0.219 0.218 0.215 0.215 0.211 0.202 0.187
## sc_grip4 0.225 0.225 0.228 0.228 0.228 0.228 0.227 0.223 0.214
## sc_grip5 0.226 0.226 0.230 0.231 0.233 0.233 0.233 0.230 0.223
## sc_grip6 0.228 0.226 0.234 0.236 0.241 0.241 0.243 0.245 0.243
## sc_grip7 0.228 0.226 0.236 0.239 0.246 0.246 0.250 0.254 0.254
## sc_grip8 0.228 0.226 0.235 0.238 0.243 0.244 0.246 0.249 0.248
## sc_grip9 0.223 0.220 0.236 0.242 0.255 0.256 0.263 0.275 0.285
## Education 0.548 0.548 0.551 0.552 0.550 0.549 0.545 0.532 0.505
## Age -0.127 -0.119 -0.171 -0.191 -0.238 -0.240 -0.272 -0.330 -0.397
## Gender -0.199 -0.201 -0.183 -0.176 -0.157 -0.156 -0.142 -0.114 -0.077
## Living_alone 0.052 0.053 0.046 0.043 0.036 0.036 0.031 0.021 0.008
## Smoking 0.064 0.065 0.060 0.058 0.053 0.053 0.049 0.041 0.031
## Apoe4 -0.002 0.000 -0.016 -0.022 -0.038 -0.039 -0.049 -0.069 -0.093
## stroke1 -0.045 -0.044 -0.051 -0.054 -0.060 -0.060 -0.064 -0.071 -0.079
## h_t1 -0.042 -0.041 -0.044 -0.045 -0.047 -0.047 -0.048 -0.050 -0.051
## dm1 -0.033 -0.032 -0.035 -0.036 -0.038 -0.038 -0.039 -0.041 -0.043
## i_Gc 0.936 0.935 0.934 0.931 0.920 0.919 0.907 0.874 0.816
## i_sc_grip 0.218 0.218 0.212 0.210 0.202 0.201 0.195 0.180 0.159
## s_Gc 0.096 0.084 0.174 0.209 0.292 0.296 0.353 0.458 0.583
## s_sc_grip -0.045 -0.049 -0.014 0.000 0.034 0.035 0.059 0.103 0.157
## sc_gr1 sc_gr2 sc_gr3 sc_gr4 sc_gr5 sc_gr6 sc_gr7 sc_gr8 sc_gr9
## Gc1
## Gc2
## Gc3
## Gc4
## Gc5
## Gc6
## Gc7
## Gc8
## Gc9
## sc_grip1 1.000
## sc_grip2 0.944 1.000
## sc_grip3 0.942 0.946 1.000
## sc_grip4 0.922 0.937 0.938 1.000
## sc_grip5 0.913 0.931 0.933 0.940 1.000
## sc_grip6 0.885 0.912 0.915 0.931 0.934 1.000
## sc_grip7 0.863 0.896 0.900 0.921 0.927 0.934 1.000
## sc_grip8 0.875 0.905 0.908 0.927 0.931 0.936 0.935 1.000
## sc_grip9 0.779 0.829 0.835 0.874 0.886 0.908 0.919 0.913 1.000
## Education 0.207 0.208 0.208 0.207 0.206 0.202 0.198 0.200 0.184
## Age -0.204 -0.242 -0.246 -0.281 -0.293 -0.319 -0.335 -0.326 -0.375
## Gender -0.788 -0.771 -0.768 -0.742 -0.730 -0.699 -0.676 -0.689 -0.592
## Living_alone -0.157 -0.159 -0.159 -0.159 -0.158 -0.156 -0.154 -0.155 -0.145
## Smoking 0.504 0.495 0.493 0.478 0.472 0.454 0.440 0.447 0.388
## Apoe4 -0.007 -0.009 -0.009 -0.010 -0.010 -0.011 -0.012 -0.012 -0.013
## stroke1 0.069 0.063 0.062 0.055 0.053 0.047 0.042 0.045 0.029
## h_t1 -0.067 -0.074 -0.075 -0.081 -0.083 -0.088 -0.090 -0.089 -0.096
## dm1 0.009 0.001 0.000 -0.008 -0.011 -0.017 -0.022 -0.019 -0.034
## i_Gc 0.227 0.235 0.236 0.241 0.242 0.243 0.243 0.243 0.238
## i_sc_grip 0.976 0.967 0.966 0.945 0.935 0.907 0.884 0.897 0.798
## s_Gc -0.037 0.009 0.014 0.059 0.076 0.112 0.136 0.124 0.206
## s_sc_grip -0.536 -0.445 -0.433 -0.336 -0.300 -0.216 -0.159 -0.189 0.016
## Eductn Age Gender Lvng_l Smokng Apoe4 strok1 h_t1 dm1
## Gc1
## Gc2
## Gc3
## Gc4
## Gc5
## Gc6
## Gc7
## Gc8
## Gc9
## sc_grip1
## sc_grip2
## sc_grip3
## sc_grip4
## sc_grip5
## sc_grip6
## sc_grip7
## sc_grip8
## sc_grip9
## Education 1.000
## Age -0.230 1.000
## Gender -0.191 -0.019 1.000
## Living_alone 0.007 0.146 0.183 1.000
## Smoking 0.120 -0.004 -0.646 -0.086 1.000
## Apoe4 -0.007 0.035 -0.006 0.015 -0.023 1.000
## stroke1 -0.017 0.129 -0.141 -0.056 0.095 -0.023 1.000
## h_t1 -0.002 0.108 0.049 0.051 -0.013 -0.070 0.087 1.000
## dm1 -0.032 0.036 -0.103 -0.021 0.074 0.025 0.021 0.102 1.000
## i_Gc 0.586 -0.135 -0.212 0.055 0.069 -0.002 -0.048 -0.044 -0.035
## i_sc_grip 0.213 -0.209 -0.807 -0.161 0.516 -0.008 0.070 -0.069 0.009
## s_Gc 0.113 -0.583 0.170 -0.068 -0.043 -0.181 -0.083 -0.034 -0.032
## s_sc_grip -0.094 -0.205 0.525 0.064 -0.321 -0.007 -0.081 -0.026 -0.068
## i_Gc i_sc_g s_Gc s_sc_g
## Gc1
## Gc2
## Gc3
## Gc4
## Gc5
## Gc6
## Gc7
## Gc8
## Gc9
## sc_grip1
## sc_grip2
## sc_grip3
## sc_grip4
## sc_grip5
## sc_grip6
## sc_grip7
## sc_grip8
## sc_grip9
## Education
## Age
## Gender
## Living_alone
## Smoking
## Apoe4
## stroke1
## h_t1
## dm1
## i_Gc 1.000
## i_sc_grip 0.233 1.000
## s_Gc 0.103 -0.038 1.000
## s_sc_grip -0.048 -0.549 0.383 1.000

## Residual Matrices

## $cov
## Gf1 Gf2 Gf3 Gf4 Gf5 Gf6 Gf7 Gf8 Gf9
## Gf1 -0.057
## Gf2 -0.061 -0.071
## Gf3 -0.039 -0.013 0.002
## Gf4 -0.006 0.015 0.075 0.102
## Gf5 -0.012 0.007 0.075 0.130 0.119
## Gf6 -0.022 -0.003 0.077 0.123 0.149 0.135
## Gf7 -0.044 -0.026 0.039 0.092 0.103 0.122 0.068
## Gf8 -0.083 -0.065 0.003 0.065 0.068 0.085 0.040 -0.027
## Gf9 -0.201 -0.227 -0.154 -0.151 -0.205 -0.213 -0.292 -0.487 -0.912
## sc_velo1 -0.076 -0.073 -0.061 -0.034 -0.057 -0.052 -0.088 -0.115 -0.281
## sc_velo2 -0.020 0.012 0.030 0.063 0.032 0.052 0.043 0.012 -0.199
## sc_velo3 -0.020 0.038 0.048 0.091 0.086 0.069 0.072 0.024 -0.181
## sc_velo4 -0.042 0.004 0.022 0.060 0.034 0.031 0.017 -0.029 -0.288
## sc_velo5 -0.015 0.027 0.052 0.104 0.088 0.090 0.078 0.029 -0.236
## sc_velo6 -0.059 0.015 0.045 0.072 0.064 0.064 0.082 -0.012 -0.281
## sc_velo7 -0.075 -0.013 0.039 0.069 0.060 0.079 0.069 -0.034 -0.429
## sc_velo8 -0.099 -0.049 -0.010 0.038 0.012 0.003 -0.015 -0.090 -0.591
## sc_velo9 -0.176 -0.170 -0.142 -0.070 -0.132 -0.113 -0.161 -0.323 -0.780
## Education -0.005 -0.003 -0.006 0.012 0.010 -0.011 -0.004 0.014 0.037
## Age 0.039 0.020 -0.015 -0.040 -0.037 -0.051 -0.039 0.019 0.206
## Gender -0.004 -0.003 0.005 -0.003 0.004 0.004 0.006 0.013 -0.023
## Living_alone 0.002 -0.001 -0.002 0.002 -0.001 -0.001 -0.003 0.009 0.008
## Smoking 0.008 -0.004 -0.004 -0.004 -0.014 0.007 0.008 -0.012 0.016
## Apoe4 0.006 0.007 -0.004 -0.016 -0.006 -0.008 0.004 0.010 0.006
## stroke1 0.004 -0.002 -0.004 -0.001 -0.002 0.005 0.003 0.002 0.013
## h_t1 0.014 -0.005 -0.011 0.002 -0.008 0.010 0.004 0.000 -0.005
## dm1 0.006 0.002 -0.009 -0.005 -0.006 -0.006 -0.003 0.002 0.010
## sc_vl1 sc_vl2 sc_vl3 sc_vl4 sc_vl5 sc_vl6 sc_vl7 sc_vl8 sc_vl9
## Gf1
## Gf2
## Gf3
## Gf4
## Gf5
## Gf6
## Gf7
## Gf8
## Gf9
## sc_velo1 -0.090
## sc_velo2 -0.057 -0.042
## sc_velo3 -0.046 0.038 0.150
## sc_velo4 -0.063 0.001 0.123 0.055
## sc_velo5 -0.075 0.013 0.130 0.185 0.181
## sc_velo6 -0.100 0.012 0.119 0.134 0.236 0.137
## sc_velo7 -0.164 -0.060 0.082 0.032 0.117 0.125 -0.117
## sc_velo8 -0.240 -0.154 0.003 -0.064 0.044 0.011 -0.108 -0.322
## sc_velo9 -0.274 -0.182 -0.042 -0.203 -0.091 -0.143 -0.322 -0.491 -0.711
## Education 0.018 -0.021 -0.017 0.009 -0.010 0.024 0.018 0.032 -0.076
## Age 0.070 -0.010 -0.054 0.019 -0.078 -0.040 0.013 0.035 0.178
## Gender 0.014 0.007 -0.006 -0.013 -0.037 -0.009 0.002 0.023 0.023
## Living_alone -0.003 0.001 0.002 -0.004 0.000 0.009 -0.002 0.005 0.012
## Smoking -0.020 -0.002 0.010 0.013 0.021 0.018 0.002 -0.032 0.002
## Apoe4 0.009 -0.006 -0.014 0.006 0.003 0.002 -0.001 0.015 -0.040
## stroke1 -0.001 0.003 0.002 -0.003 -0.002 -0.012 0.003 -0.011 0.036
## h_t1 0.007 -0.005 0.001 0.008 -0.006 -0.011 -0.010 -0.032 0.077
## dm1 0.003 -0.006 -0.002 -0.001 0.004 0.012 0.004 0.012 -0.019
## Eductn Age Gender Lvng_l Smokng Apoe4 strok1 h_t1 dm1
## Gf1
## Gf2
## Gf3
## Gf4
## Gf5
## Gf6
## Gf7
## Gf8
## Gf9
## sc_velo1
## sc_velo2
## sc_velo3
## sc_velo4
## sc_velo5
## sc_velo6
## sc_velo7
## sc_velo8
## sc_velo9
## Education 0.000
## Age 0.000 0.000
## Gender 0.000 0.000 0.000
## Living_alone 0.000 0.000 0.000 0.000
## Smoking 0.000 0.000 0.000 0.000 0.000
## Apoe4 0.000 0.000 0.000 0.000 0.000 0.000
## stroke1 0.000 0.000 0.000 0.000 0.000 0.000 0.000
## h_t1 0.000 0.000 0.000 0.000 0.000 0.000 0.000 0.000
## dm1 0.000 0.000 0.000 0.000 0.000 0.000 0.000 0.000 0.000
##
## $mean
## Gf1 Gf2 Gf3 Gf4 Gf5 Gf6
## -0.032 -0.020 0.006 0.013 -0.001 0.012
## Gf7 Gf8 Gf9 sc_velo1 sc_velo2 sc_velo3
## 0.003 -0.001 0.265 -0.054 -0.001 0.015
## sc_velo4 sc_velo5 sc_velo6 sc_velo7 sc_velo8 sc_velo9
## -0.009 0.027 0.012 -0.011 -0.001 0.142
## Education Age Gender Living_alone Smoking Apoe4
## 0.000 0.000 0.000 0.000 0.000 0.000
## stroke1 h_t1 dm1
## 0.000 0.000 0.000
##
## $cov
## Gf1 Gf2 Gf3 Gf4 Gf5 Gf6 Gf7 Gf8 Gf9
## Gf1 -0.059
## Gf2 -0.061 -0.072
## Gf3 -0.039 -0.012 0.001
## Gf4 -0.008 0.014 0.075 0.100
## Gf5 -0.014 0.008 0.077 0.131 0.122
## Gf6 -0.023 -0.001 0.078 0.127 0.155 0.141
## Gf7 -0.044 -0.023 0.039 0.097 0.111 0.132 0.081
## Gf8 -0.089 -0.062 0.006 0.075 0.083 0.105 0.067 0.018
## Gf9 -0.186 -0.206 -0.139 -0.121 -0.161 -0.184 -0.249 -0.436 -0.816
## sc_grip1 -0.018 -0.011 -0.008 0.021 0.022 0.017 -0.010 -0.030 0.065
## sc_grip2 -0.010 0.003 -0.001 0.015 0.013 0.010 -0.013 -0.038 0.025
## sc_grip3 -0.008 -0.002 0.010 0.023 0.030 0.029 0.008 -0.013 0.041
## sc_grip4 -0.013 -0.001 0.018 0.025 0.025 0.015 -0.002 -0.025 -0.014
## sc_grip5 -0.030 -0.027 -0.017 -0.001 -0.004 0.002 -0.019 -0.046 -0.071
## sc_grip6 -0.030 -0.021 -0.009 0.003 0.005 0.019 -0.016 -0.037 -0.126
## sc_grip7 -0.028 -0.015 0.002 0.014 0.016 0.030 0.016 -0.015 -0.097
## sc_grip8 0.030 0.056 0.079 0.107 0.100 0.111 0.096 0.100 -0.030
## sc_grip9 -0.039 -0.036 -0.003 -0.003 -0.030 -0.026 -0.047 -0.117 -0.187
## Education -0.004 -0.003 -0.007 0.011 0.010 -0.011 -0.003 0.006 0.058
## Age 0.040 0.020 -0.016 -0.043 -0.039 -0.052 -0.047 0.002 0.214
## Gender -0.002 -0.004 0.005 -0.003 0.003 0.002 0.003 0.011 -0.050
## Living_alone 0.003 -0.001 -0.002 0.001 -0.002 -0.002 -0.001 0.008 -0.002
## Smoking 0.005 -0.004 -0.004 -0.002 -0.012 0.012 0.015 -0.007 0.065
## Apoe4 0.005 0.008 -0.004 -0.016 -0.005 -0.008 0.003 0.012 0.020
## stroke1 0.005 -0.002 -0.004 -0.001 -0.003 0.003 0.002 0.001 0.012
## h_t1 0.014 -0.005 -0.011 0.003 -0.008 0.011 0.006 0.005 -0.002
## dm1 0.006 0.001 -0.009 -0.005 -0.007 -0.006 -0.004 -0.001 0.002
## sc_gr1 sc_gr2 sc_gr3 sc_gr4 sc_gr5 sc_gr6 sc_gr7 sc_gr8 sc_gr9
## Gf1
## Gf2
## Gf3
## Gf4
## Gf5
## Gf6
## Gf7
## Gf8
## Gf9
## sc_grip1 0.030
## sc_grip2 0.011 -0.011
## sc_grip3 -0.013 -0.019 -0.024
## sc_grip4 0.005 -0.023 -0.008 -0.008
## sc_grip5 0.019 -0.015 -0.005 0.014 0.011
## sc_grip6 -0.005 -0.030 -0.026 -0.017 -0.002 -0.048
## sc_grip7 0.019 -0.015 -0.002 0.006 0.018 -0.004 -0.006
## sc_grip8 -0.001 -0.032 -0.002 0.005 0.009 0.003 0.032 0.069
## sc_grip9 0.113 0.059 0.079 0.046 0.053 0.006 0.022 0.049 0.022
## Education -0.002 0.003 -0.002 0.012 -0.016 -0.022 0.008 0.020 0.027
## Age -0.006 0.008 0.005 0.000 -0.001 0.011 -0.001 -0.075 -0.008
## Gender -0.007 0.003 0.008 0.004 -0.004 0.004 -0.003 0.010 -0.029
## Living_alone -0.002 0.002 -0.001 0.001 0.002 0.000 -0.001 0.001 0.001
## Smoking 0.011 -0.001 -0.017 -0.012 0.005 -0.002 0.010 -0.002 0.044
## Apoe4 0.001 0.002 -0.004 0.000 -0.003 -0.003 0.009 0.001 -0.001
## stroke1 -0.001 0.002 -0.001 -0.002 0.000 0.001 0.000 -0.006 0.005
## h_t1 0.001 0.003 -0.002 -0.002 0.005 -0.001 0.009 -0.001 0.000
## dm1 -0.003 0.003 0.003 -0.003 0.005 -0.005 -0.001 -0.007 -0.009
## Eductn Age Gender Lvng_l Smokng Apoe4 strok1 h_t1 dm1
## Gf1
## Gf2
## Gf3
## Gf4
## Gf5
## Gf6
## Gf7
## Gf8
## Gf9
## sc_grip1
## sc_grip2
## sc_grip3
## sc_grip4
## sc_grip5
## sc_grip6
## sc_grip7
## sc_grip8
## sc_grip9
## Education 0.000
## Age 0.000 0.000
## Gender 0.000 0.000 0.000
## Living_alone 0.000 0.000 0.000 0.000
## Smoking 0.000 0.000 0.000 0.000 0.000
## Apoe4 0.000 0.000 0.000 0.000 0.000 0.000
## stroke1 0.000 0.000 0.000 0.000 0.000 0.000 0.000
## h_t1 0.000 0.000 0.000 0.000 0.000 0.000 0.000 0.000
## dm1 0.000 0.000 0.000 0.000 0.000 0.000 0.000 0.000 0.000
##
## $mean
## Gf1 Gf2 Gf3 Gf4 Gf5 Gf6
## -0.027 -0.017 0.005 0.008 -0.005 0.009
## Gf7 Gf8 Gf9 sc_grip1 sc_grip2 sc_grip3
## -0.005 -0.012 0.303 -0.004 -0.012 0.016
## sc_grip4 sc_grip5 sc_grip6 sc_grip7 sc_grip8 sc_grip9
## 0.003 0.001 0.003 -0.006 -0.032 0.023
## Education Age Gender Living_alone Smoking Apoe4
## 0.000 0.000 0.000 0.000 0.000 0.000
## stroke1 h_t1 dm1
## 0.000 0.000 0.000
##
## $cov
## Gc1 Gc2 Gc3 Gc4 Gc5 Gc6 Gc7 Gc8 Gc9
## Gc1 -0.019
## Gc2 -0.024 -0.033
## Gc3 -0.014 0.003 -0.008
## Gc4 -0.002 0.002 0.031 0.042
## Gc5 -0.018 0.005 0.027 0.052 0.046
## Gc6 -0.025 -0.007 0.007 0.028 0.047 0.012
## Gc7 -0.005 0.010 0.034 0.047 0.059 0.044 0.072
## Gc8 -0.037 0.012 -0.006 0.028 0.010 0.014 0.037 0.004
## Gc9 -0.030 -0.015 -0.025 0.006 -0.004 0.000 0.004 -0.003 -0.006
## sc_velo1 0.002 0.015 -0.006 0.006 0.008 0.009 -0.014 -0.001 0.014
## sc_velo2 -0.016 -0.011 -0.007 -0.010 0.005 0.015 0.000 0.002 0.036
## sc_velo3 -0.028 -0.031 -0.013 -0.007 0.006 0.005 -0.017 0.023 0.019
## sc_velo4 -0.005 0.009 0.007 0.027 0.050 0.043 -0.006 0.041 0.060
## sc_velo5 -0.028 -0.021 -0.021 0.020 0.049 0.041 0.005 0.041 0.089
## sc_velo6 -0.024 -0.010 -0.024 0.017 0.035 0.040 -0.010 0.044 0.074
## sc_velo7 -0.023 0.013 -0.004 0.028 0.046 0.038 0.003 0.026 0.055
## sc_velo8 -0.012 0.045 -0.004 0.062 0.080 0.057 -0.001 0.055 0.074
## sc_velo9 -0.066 0.001 -0.015 -0.043 0.053 0.047 -0.035 -0.035 0.010
## Education 0.000 -0.016 0.005 0.010 0.025 -0.006 0.017 -0.014 0.021
## Age 0.028 0.000 -0.006 -0.007 -0.017 -0.022 -0.016 -0.025 -0.089
## Gender -0.001 -0.002 -0.001 0.007 0.006 -0.004 -0.014 0.000 0.001
## Living_alone -0.001 0.001 -0.002 0.001 0.005 -0.005 0.005 -0.006 -0.009
## Smoking 0.009 -0.001 0.002 -0.017 -0.013 0.007 0.013 0.000 -0.009
## Apoe4 -0.003 0.006 0.001 -0.009 0.004 -0.010 -0.002 0.002 0.010
## stroke1 -0.001 0.002 0.000 -0.003 -0.002 0.000 0.004 0.001 -0.021
## h_t1 0.001 0.000 -0.001 0.001 0.001 0.006 -0.005 -0.008 -0.008
## dm1 0.003 -0.001 0.001 0.004 -0.010 -0.005 0.008 -0.004 -0.006
## sc_vl1 sc_vl2 sc_vl3 sc_vl4 sc_vl5 sc_vl6 sc_vl7 sc_vl8 sc_vl9
## Gc1
## Gc2
## Gc3
## Gc4
## Gc5
## Gc6
## Gc7
## Gc8
## Gc9
## sc_velo1 -0.095
## sc_velo2 -0.063 -0.052
## sc_velo3 -0.049 0.030 0.144
## sc_velo4 -0.059 0.003 0.127 0.066
## sc_velo5 -0.073 0.013 0.133 0.198 0.191
## sc_velo6 -0.088 0.019 0.135 0.160 0.260 0.171
## sc_velo7 -0.158 -0.063 0.095 0.052 0.134 0.161 -0.076
## sc_velo8 -0.219 -0.130 0.048 -0.018 0.095 0.080 -0.026 -0.185
## sc_velo9 -0.234 -0.105 0.042 -0.090 0.017 -0.024 -0.166 -0.255 -0.300
## Education 0.019 -0.022 -0.021 0.007 -0.013 0.025 0.021 0.035 -0.057
## Age 0.071 -0.009 -0.055 0.017 -0.079 -0.043 0.001 0.007 0.105
## Gender 0.014 0.005 -0.003 -0.013 -0.035 -0.008 -0.002 0.016 -0.001
## Living_alone -0.002 0.001 0.002 -0.004 -0.001 0.006 -0.004 0.005 0.001
## Smoking -0.020 -0.002 0.008 0.012 0.020 0.017 0.008 -0.017 0.018
## Apoe4 0.009 -0.007 -0.014 0.006 0.004 0.006 -0.002 0.011 -0.061
## stroke1 -0.001 0.004 0.002 -0.004 -0.004 -0.012 0.002 -0.012 0.043
## h_t1 0.006 -0.005 0.002 0.008 -0.008 -0.014 -0.017 -0.039 0.084
## dm1 0.003 -0.006 -0.001 0.000 0.005 0.014 0.003 0.007 -0.023
## Eductn Age Gender Lvng_l Smokng Apoe4 strok1 h_t1 dm1
## Gc1
## Gc2
## Gc3
## Gc4
## Gc5
## Gc6
## Gc7
## Gc8
## Gc9
## sc_velo1
## sc_velo2
## sc_velo3
## sc_velo4
## sc_velo5
## sc_velo6
## sc_velo7
## sc_velo8
## sc_velo9
## Education 0.000
## Age 0.000 0.000
## Gender 0.000 0.000 0.000
## Living_alone 0.000 0.000 0.000 0.000
## Smoking 0.000 0.000 0.000 0.000 0.000
## Apoe4 0.000 0.000 0.000 0.000 0.000 0.000
## stroke1 0.000 0.000 0.000 0.000 0.000 0.000 0.000
## h_t1 0.000 0.000 0.000 0.000 0.000 0.000 0.000 0.000
## dm1 0.000 0.000 0.000 0.000 0.000 0.000 0.000 0.000 0.000
##
## $mean
## Gc1 Gc2 Gc3 Gc4 Gc5 Gc6
## -0.048 0.007 -0.010 0.038 0.010 0.036
## Gc7 Gc8 Gc9 sc_velo1 sc_velo2 sc_velo3
## 0.016 -0.006 -0.263 -0.050 -0.002 0.017
## sc_velo4 sc_velo5 sc_velo6 sc_velo7 sc_velo8 sc_velo9
## -0.009 0.031 0.013 -0.017 -0.040 0.043
## Education Age Gender Living_alone Smoking Apoe4
## 0.000 0.000 0.000 0.000 0.000 0.000
## stroke1 h_t1 dm1
## 0.000 0.000 0.000
##
## $cov
## Gc1 Gc2 Gc3 Gc4 Gc5 Gc6 Gc7 Gc8 Gc9
## Gc1 -0.020
## Gc2 -0.025 -0.034
## Gc3 -0.017 0.000 -0.013
## Gc4 -0.002 0.003 0.030 0.044
## Gc5 -0.020 0.004 0.024 0.054 0.048
## Gc6 -0.027 -0.006 0.006 0.032 0.051 0.018
## Gc7 -0.008 0.008 0.029 0.047 0.059 0.047 0.069
## Gc8 -0.040 0.009 -0.007 0.028 0.010 0.016 0.035 -0.003
## Gc9 -0.044 -0.025 -0.040 0.001 -0.012 -0.008 -0.008 -0.015 -0.035
## sc_grip1 -0.007 0.005 0.008 -0.021 0.001 0.018 0.040 0.001 -0.010
## sc_grip2 -0.006 0.002 0.014 -0.014 0.006 0.026 0.042 0.008 0.010
## sc_grip3 -0.013 -0.005 0.003 -0.025 0.002 0.008 0.034 -0.008 0.010
## sc_grip4 -0.001 0.013 0.021 -0.007 0.014 0.030 0.041 0.010 0.022
## sc_grip5 -0.023 -0.014 -0.008 -0.036 -0.012 0.002 0.017 -0.018 -0.001
## sc_grip6 -0.027 -0.022 -0.004 -0.030 -0.015 0.001 0.012 -0.010 -0.011
## sc_grip7 -0.009 0.007 0.017 0.001 0.012 0.023 0.044 0.017 0.029
## sc_grip8 0.006 0.025 0.032 0.031 0.033 0.042 0.059 0.051 0.080
## sc_grip9 0.015 0.020 0.033 0.010 0.024 0.033 0.042 0.036 0.062
## Education -0.001 -0.016 0.002 0.011 0.027 -0.006 0.016 -0.017 0.004
## Age 0.031 0.000 -0.006 -0.008 -0.022 -0.026 -0.018 -0.016 -0.068
## Gender -0.001 -0.002 -0.001 0.005 0.006 -0.004 -0.015 -0.001 -0.005
## Living_alone -0.001 0.001 -0.002 0.000 0.005 -0.005 0.004 -0.006 -0.009
## Smoking 0.009 -0.002 0.002 -0.015 -0.012 0.009 0.018 0.000 0.004
## Apoe4 -0.002 0.005 0.001 -0.009 0.003 -0.009 0.000 0.002 0.013
## stroke1 -0.001 0.002 0.000 -0.002 -0.002 -0.001 0.004 0.002 -0.017
## h_t1 0.001 0.000 -0.001 0.000 0.000 0.004 -0.004 -0.007 -0.008
## dm1 0.003 -0.001 0.000 0.003 -0.011 -0.006 0.005 -0.003 -0.011
## sc_gr1 sc_gr2 sc_gr3 sc_gr4 sc_gr5 sc_gr6 sc_gr7 sc_gr8 sc_gr9
## Gc1
## Gc2
## Gc3
## Gc4
## Gc5
## Gc6
## Gc7
## Gc8
## Gc9
## sc_grip1 0.027
## sc_grip2 0.009 -0.012
## sc_grip3 -0.015 -0.020 -0.025
## sc_grip4 0.005 -0.021 -0.006 -0.006
## sc_grip5 0.019 -0.014 -0.004 0.017 0.013
## sc_grip6 -0.004 -0.028 -0.023 -0.012 0.003 -0.041
## sc_grip7 0.022 -0.012 0.001 0.012 0.024 0.003 0.003
## sc_grip8 -0.005 -0.034 -0.003 0.004 0.006 0.002 0.034 0.062
## sc_grip9 0.105 0.055 0.076 0.052 0.057 0.016 0.039 0.059 0.046
## Education -0.002 0.003 -0.001 0.012 -0.016 -0.023 0.011 0.022 0.025
## Age -0.005 0.008 0.005 0.000 -0.002 0.009 -0.006 -0.073 -0.032
## Gender -0.006 0.003 0.008 0.003 -0.004 0.004 -0.004 0.008 -0.023
## Living_alone -0.001 0.002 -0.001 0.001 0.001 0.000 -0.001 0.001 -0.006
## Smoking 0.011 -0.002 -0.017 -0.011 0.005 -0.002 0.011 0.001 0.032
## Apoe4 0.001 0.002 -0.004 -0.001 -0.003 -0.003 0.008 0.004 -0.001
## stroke1 -0.001 0.002 -0.001 -0.002 0.000 -0.001 -0.001 -0.005 -0.004
## h_t1 0.000 0.003 -0.002 -0.002 0.005 -0.001 0.005 -0.004 -0.004
## dm1 -0.003 0.003 0.003 -0.003 0.004 -0.006 -0.002 -0.007 -0.014
## Eductn Age Gender Lvng_l Smokng Apoe4 strok1 h_t1 dm1
## Gc1
## Gc2
## Gc3
## Gc4
## Gc5
## Gc6
## Gc7
## Gc8
## Gc9
## sc_grip1
## sc_grip2
## sc_grip3
## sc_grip4
## sc_grip5
## sc_grip6
## sc_grip7
## sc_grip8
## sc_grip9
## Education 0.000
## Age 0.000 0.000
## Gender 0.000 0.000 0.000
## Living_alone 0.000 0.000 0.000 0.000
## Smoking 0.000 0.000 0.000 0.000 0.000
## Apoe4 0.000 0.000 0.000 0.000 0.000 0.000
## stroke1 0.000 0.000 0.000 0.000 0.000 0.000 0.000
## h_t1 0.000 0.000 0.000 0.000 0.000 0.000 0.000 0.000
## dm1 0.000 0.000 0.000 0.000 0.000 0.000 0.000 0.000 0.000
##
## $mean
## Gc1 Gc2 Gc3 Gc4 Gc5 Gc6
## -0.048 0.004 -0.010 0.039 0.008 0.032
## Gc7 Gc8 Gc9 sc_grip1 sc_grip2 sc_grip3
## 0.014 -0.002 -0.243 -0.004 -0.011 0.017
## sc_grip4 sc_grip5 sc_grip6 sc_grip7 sc_grip8 sc_grip9
## 0.004 0.002 0.003 -0.015 -0.027 -0.060
## Education Age Gender Living_alone Smoking Apoe4
## 0.000 0.000 0.000 0.000 0.000 0.000
## stroke1 h_t1 dm1
## 0.000 0.000 0.000
